# Differential Exposure to Hazardous Air Pollution in the United States: A Multilevel Analysis of Urbanization and Neighborhood Socioeconomic Deprivation

**DOI:** 10.3390/ijerph9062204

**Published:** 2012-06-13

**Authors:** Gary S. Young, Mary A. Fox, Michael Trush, Norma Kanarek, Thomas A. Glass, Frank C. Curriero

**Affiliations:** 1 Department of Environmental Health Sciences, Johns Hopkins University, Bloomberg School of Public Health, 615 North Wolfe St., Baltimore, MD 21205, USA; Email: mtrush@jhsph.edu (M.T.); nkanarek@jhsph.edu (N.K.); fcurrier@jhsph.edu (F.C.C.); 2 Department of Health Policy and Management, Johns Hopkins University, Bloomberg School of Public Health, 624 N. Broadway, Baltimore, MD 21205, USA; Email: mfox@jhsph.edu; 3 Department of Epidemiology, Johns Hopkins University, Bloomberg School of Public Health, 615 North Wolfe St., Baltimore, MD 21205, USA; Email: tglass@jhsph.edu

**Keywords:** cumulative risk assessment, socioeconomic deprivation, hazardous air pollution, respiratory health exposure hazards, social epidemiology, environmental epidemiology

## Abstract

Population exposure to multiple chemicals in air presents significant challenges for environmental public health. Air quality regulations distinguish criteria air pollutants (CAPs) (e.g., ozone, PM2.5) from hazardous air pollutants (HAPs)—187 chemicals which include carcinogens and others that are associated with respiratory, cardiovascular, neurological and numerous other non-cancer health effects. Evidence of the public’s cumulative exposure and the health effects of HAPs are quite limited. A multilevel model is used to assess differential exposure to HAP respiratory, neurological, and cancer hazards (2005) related to the Townsend Index of Socioeconomic Deprivation (TSI), after adjustment for regional population size and economic activity, and local population density. We found significant positive associations between tract TSI and respiratory and cancer HAP exposure hazards, and smaller effects for neurological HAPs. Tracts in the top quintile of TSI have between 38%–60% higher HAP exposure than the bottom quintile; increasing population size from the bottom quintile to the top quintile modifies HAP exposure hazard related to TSI, increasing cancer HAP exposure hazard by 6% to 20% and increasing respiratory HAP exposure hazard by 12% to 27%. This study demonstrates the value of social epidemiological methods for analyzing differential exposure and advancing cumulative risk assessment.

## 1. Introduction

Air pollution is a major environmental public health issue in the United States directly affecting wellbeing and quality of life. Numerous studies attribute excess respiratory and cardiovascular morbidity and higher mortality rates to air pollution, particularly among urbanized places [1,2,3,4,5,6,7,8,9,10,11,12,13,14,15,16,17,18,19,20,21,22,23,24,25,26,27,28,29,30,31,32,33,34]. Air quality regulations in the United States from the beginning of the Clean Air Act in the early 1970s have separated criteria air pollutants (CAPs), which are ubiquitous chemicals in the environment (e.g., sulfur dioxide, ozone, and particulate matter (2.5 μm), from hazardous air pollutants (HAPs). HAPs comprise 187 chemicals including arsenic, lead, cadmium, pollutant gases, solvents, and pesticides many of which are thought to have no minimum threshold of exposure that can be considered safe [35]. While many State Implementation Plans employed under the Clean Air Act have made progress in reducing CAPs and other chemicals regulated as HAPs, evidence on environmental exposure to HAPs or their associated health effects is quite limited [36,37,38].

While acute exposures to high concentrations to HAPs are clearly dangerous, the cumulative health effects of low level chronic exposure are uncertain, given the multiple organs targeted by these metals and chemicals. Point source HAP emissions typically involve small amounts of dozens of chemicals, each with potentially different health effects based on the organ targeted by the chemical. For many of these chemicals small exposures are biologically capable of significant health effects. Similar to CAPs, ambient air exposure to HAPs is typically concentrated in urban and industrial areas and among adjacent populations. 

HAPs have been problematic for both risk assessment methods and risk management policy. The cumulative burden of air pollution exposure among residential communities adjacent to industrial land use and point source emissions presents significant environmental justice policy issues for air quality management, and scientific challenges for risk assessment. One of the major challenges in environmental epidemiology and risk assessment in urban settings is the confounding of health effects associated with poverty with those related to environmental exposure. Complicating differential exposure among those of lower socioeconomic position is the potential for differential susceptibility to chemical exposures. The National Academy of Sciences has challenged the adequacy of risk assessment methods due to the potential for differential susceptibility, and cites the need to improve our understanding of population vulnerability to chemical exposure, particularly among populations subject to high levels of *nonchemical* stressors such as socioeconomic deprivation (SED) [39].

This paper seeks to contribute to risk assessment methods by demonstrating the value of incorporating social epidemiology in analyzing social gradients in HAP exposure related to urbanization and neighborhood SED. Developing better metrics and dose-response models relating to social gradients in exposure and health outcomes, particularly among vulnerable populations, can help advance cumulative risk assessment.

The study uses national emissions inventory data as modeled in the National Air Toxics Assessment in 2005 and includes health hazard exposure indices for chemicals related to risk of cancer risk and respiratory and neurological noncancerous health endpoints. We used a social determinant of health framework to develop a multilevel model to analyze how the *baseline range HAP exposure hazards* shifts with *level of urbanization* and related *economic activity* and degree of SED at the local (neighborhood) level. Urbanization is conceptualized as the concentration of residential population *and* economic activity, and is measured by local census tract population density, and tracts’ respective county population size, number employed and aggregate wage earned the tracts’ host county. The Townsend Index of Socioeconomic Deprivation (TSI) is used to measure SED at the census tract level [40]. Assessment of differential exposure relating to SED is strengthened by including known or suspected confounders and covariates relating to HAPs.

This study conceptualizes variation in local neighborhood chemical exposure to be decisively influenced by the population size in the region (with Host County of census tract as proxy) in combination with the economic activity generated in region (host county business metrics as proxy). How SED is generated and how it is geographically distribution SED is complicated [40,41,42,43]. While there is substantial rural poverty in the USA, greater numbers of households with SED are located in urban areas, often in ‘inner cities’. The key question is whether SED is confounded with HAP because of their common urban context, or whether SED works to modify HAP exposure.

Accordingly, in this study we tested the following hypotheses:

Hypothesis 1: The greater the population size and level of regional economic activity, the higher the level of HAP exposure hazard in the region.
Hypothesis 2: The greater the population size and level of regional economic activity, the higher the level of socioeconomic deprivation among the local neighborhoods.
Hypothesis 3: The higher the level of neighborhood socioeconomic deprivation, the higher the level of HAP exposure, after adjustment for population size and level of economic activity.

Hypothesis 1 identifies population size and level of economic activity as primary drivers of HAP exposure hazard. The second hypothesis addresses the necessary condition for SED to be confounded with HAP exposure hazard: their common relationship with the urban concentration of population and level of economic activity. As an alternative to a spurious relationship between SED and HAP exposure hazard, hypothesis 3 presents a SED differential exposure model. The geographic concentration of HAP exposure and higher SED households, it is argued, is related to “disinvestment” and public policies that work as a form of disenfranchisement among those that live in economically and politically weak urban places. In this model, neighborhood SED is directly related to HAP exposure after adjustment for urban concentration or level of economic activity. 

This study evaluated evidence of “differential exposure” of HAP exposure hazard related to neighborhood SED, independent of U.S. geographic region and level of urbanization in terms of population size, density and regional economic activities. This research addresses the question: how does level of HAP respiratory, neurological, and cancer exposure hazard vary by regional population size and level of economic activity and local population density and SED. Is there evidence of differential HAP exposure related to localized SED, independent of the regional context of population level and economic activity, or is SED confounded with urbanization and HAP exposure? Among the unique contributions of this study are the application of a multilevel exposure model that examines the relationship of HAP exposure and neighborhood SED after adjustment for the level of urbanization, population density, and the regional level of economic activity that is monitored at the county level. 

## 2. Research Design and Methods

The study design involves a retrospective, cross-sectional, population-based analysis of HAP exposure at the census tract level in 2005. Predictors of HAP exposure include census-tract neighborhood SED and urbanization as measured by population density at the census tract level, and two regional variables—county population size and county business activity. The study population consists of the 65,166 census tracts (99.6% of the universe of 65,443) and their respective 3121 United States (U.S.) counties (99.4% of the universe of 3141 counties) for which there is complete data available. 

**Figure 1:**
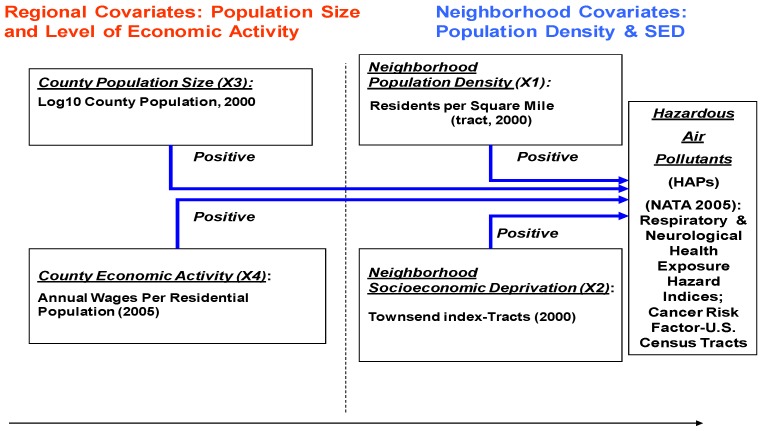
Multilevel Urban Market Exposure Model for HAPs: Differential Exposure Related to Neighborhood Socioeconomic Deprivation.

We assembled three databases to permit the specification of a multilevel HAP differential exposure model based on census tract, county, and regional criteria: (a) the National Air Toxics Assessment (NATA) HAP exposure data relating to health hazard exposure measures at the census tract level; (b) decennial census data (2000) on tract characteristics relating to population density and socioeconomic deprivation, and related demographics for tracts’ host counties, *i.e.*, regional population; and (c) County Business Pattern survey data for all U.S. counties in 2005 on number employed and annual wages. Regarding the geographic dimension of the analysis, we used the Census Bureau’s categorization of States into nine regions to profile HAP exposure. Figure 1 summarizes the variables and indicators included in the multilevel exposure model used in this study. 

We examined the variation in modeled HAP exposure estimates from the NATA data regarding respiratory, neurological, and cancer-related exposure hazard indices and cancer risk factors, and give particular attention to the magnitude and upper tail of the distribution of HAP exposure: first at progressive deciles of census tracts related to population size, density, economic activity, and the Townsend Index, to better understand the social gradient of HAP exposure in the United States unadjusted for covariates; and then we tested for differential HAP exposure related to tract SED after adjustment for tract and county population size, density, and economic activity focusing on three HAP hazard exposure metrics (respiratory, neurological, and cancer) for 2005 using random intercept mixed models with maximum likelihood model estimation procedures. 

### 2.1. Assessment of HAP Exposure

The U.S. Environmental Protection Agency (EPA) has been conducting a NATA and providing modeled estimates of ambient air concentration of progressively larger subsets of the chemicals classified as HAPs for 1999, 2002, and 2005, which will be the focus of this study. Among noncancer health endpoints, the U.S. EPA includes calculations of respiratory and neurological health hazard indices that quantify the ambient air concentration of selected HAPs targeting the lung and neurological functions, as the sum of chemical–specific health effects targeting a specific organ (e.g., the lung) [44]. These risk estimates are surrounded by substantial uncertainties associated with characterizing sources, exposures, and pollutant hazards, whereas cancer risk estimates are calculated with slope factors in toxicological databases for selected known or suspected carcinogens.

### 2.2. Measurement of Socioeconomic Deprivation

This paper focuses on the Townsend Index as the measure of SED [40]. Prior to specification of the Townsend Index, we conducted a sensitivity analysis of alternative measures of population size, economic activity and SED using the Index of Neighborhood Concentrated Disadvantage in contrast to the Townsend Index. We evaluated number employed and aggregate payroll of those employed with the respective county. All measures tested had significant positive relationships with HAP exposure hazards, however, the *Townsend Index* and *Average Wages per Employee* were more robust indicators (*i.e.*, smaller variation) and thus thought to provide stronger tests of the potency of SED.

The four variables that comprise the TSI are:

- Unemployment as a percentage of those aged 16 and over who are economically active.
- Households without access to a car (car lease or ownership), as a percentage of all households.
- Residential household renting, as a percentage of all households.
- Percentage of households with “crowded housing,” *i.e.*, the number of residents exceeds the number of rooms within the household.

The combination of each census tract’s respective standard deviates (or z scores) for these four variables forms the SED score for each tract, and represents its cumulative deviance from the average, positive or negative, among the universe of census tracts. The higher the Townsend Index score, the more deprived and disadvantaged an area is thought to be. 

### 2.3. Measures of Urbanization and Economic Activity

This study focused on measures of population density at the census tract level and population size at the county level as a means of measuring level of urbanization. 

The ***County Business Patterns***database provides the only source of annual, complete, and consistent county-level data for U.S. business establishments, with industry detail. Economic activity is measured by average annual wages per employee reported in the 2005 County Business Survey.

### 2.4. Multilevel Statistical Modeling

**Figure 2:**
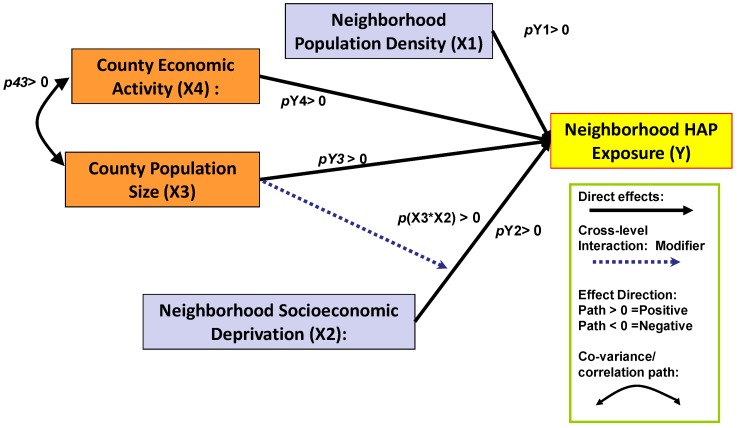
Multilevel HAP Exposure Model: County-Level Population Size as Effect Modifier of Tract-Level Socioeconomic Deprivation on HAP Exposure Risk.

Figure 2 provides a view of the interrelationship of SED with HAP exposure and the regional population level-measured as county population—and the economic activity in the region. The figure illustrates four distinct direct effects—positive relationships between HAP exposure hazard and neighborhood (tract) population density, neighborhood SED (tract Townsend Index), and county population size and level of economic activity. The key issue remains whether SED is a confounder or effect modifier of the relationship of SED with HAP exposure. The diagram includes an interaction term signified by the pathway from county population size to SED. The empirical evidence in support of SED as a confounder *versus* an effect modifier will be found in the size, direction, and statistical significance of the pathway ***p***(X3*X2), representing a conjoint effect of county population size and tract SED. 

*Multilevel HAP Exposure Equation*

𝒀ij = γ_00 _+ γ_10 _(Tract Population Density _ij_)    TRACT LEVEL FIXED EFFECTS

 + γ_11 _(Tract Socioeconomic Deprivation _ij_) 

 +γ_01 _(County Population Size _j_)    COUNTY LEVEL FIXED EFFECTS

 + γ_02 _(County Economic Activity _j_)    CROSSLEVEL INTERACTION

 + γ_12 _(County Population Size _j _*Tract Socioeconomic Deprivation _ij_)

 + u_0j_ + u_0j _(Tract Socioeconomic Deprivation _ij_) 

 + r_ij _    COUNTY LEVEL RANDOM INTERCEPT

 +γ_03 _(New England Region _j_)    REGIONAL LEVEL FIXED EFFECTS

 +γ_04 _(Middle Atlantic Region _j_)    (Note: Mountain Region is excluded

 +γ_05 _(East North Central Region _j_)    and thus is reflected in the intercept) +γ_06 _(West North Central Region _j_) 

 +γ_07 _(South Atlantic Region _j_) 

 +γ_08 _(East South Central Region _j_) 

 +γ_09 _(West South Central Region _j_) 

 +γ_0 10 _(Pacific Region _j_) 

It should be noted that the models’ explanation of the relationship of SED with HAP exposure represents a “state of exposure” that *would be expected in the absence of aggressive HAP risk management policies at the State or local level*. In practice, there are significant variations in state environmental policies regarding risk management of HAPs, as well as variation in the degree to which such policies are designed to meliorate disproportionate exposure burden among geographically concentrated vulnerable populations. The prevalence and success of such policies would be expected to work at attenuating the relationship of HAP exposure and SED. Nevertheless, this study advocates there is value in assessing the “signal” (*i.e.*, covariate) representing the relationship between SED and HAP exposure at a national level, and attempts to adjust for the policy environment by introducing geographic region into the model.

Inclusion of geographic region in a multilevel model, adjusting for urbanization and economic activity and neighborhood SED, provides a means for *improving* the estimation of the model’s coefficients. It does this by reducing the model’s residual error by accounting for the spatial dependency of unexamined confounders of SED and HAPs. Most significant for this study would be adjustment for HAP enforcement policies which share certain commonalities among different regions in the United States. Equation (1) identifies the parameters of the model which we used to estimate HAP respiratory and neurological health hazards indices and cancer risk factor for 2005 using STATA version 11 [45].

## 3. Results

Table 1 shows descriptive statistics for population size, economic activity, TSI and exposure data used in this study. A breakdown of the U.S. population in 2000, by census tracts and counties according to geographic census division, is provided in Table A1 in the Appendix. Census tracts, as a geographic unit, are distributed disproportionately among urban counties; they are administrative units designed for the purpose of counting the population and conducting sample surveys of the population’s characteristics. 

The contrast in the distribution in social and economic characteristics between tracts and counties illustrates that most census tracts are in urban areas while counties are predominantly rural. In spite of the skewed geographic distribution of the population in census tracts, there remains variation of economic activity both within and among urban and rural counties.

### 3.1. Variation in HAP Exposure Risk

**Figure 3:**
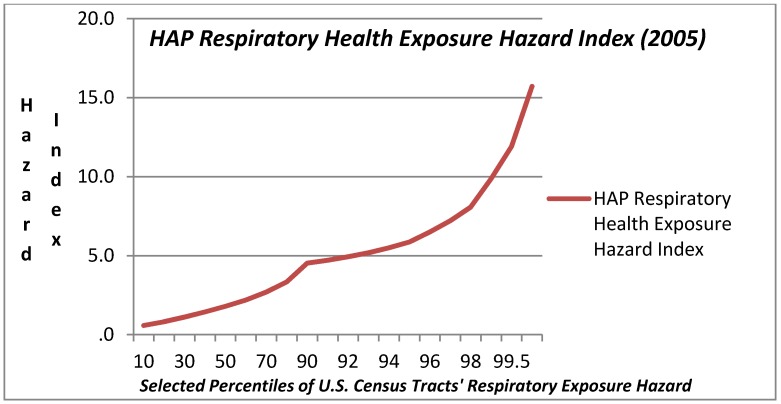
Hazardous Air Pollution (HAP) Respiratory Health Exposure Hazard Index (2005) Values at Selected Percentiles for U.S. Census Tracts (U.S. E.P.A. National Air Toxics Assessment, 2005) [44].

**Figure 4:**
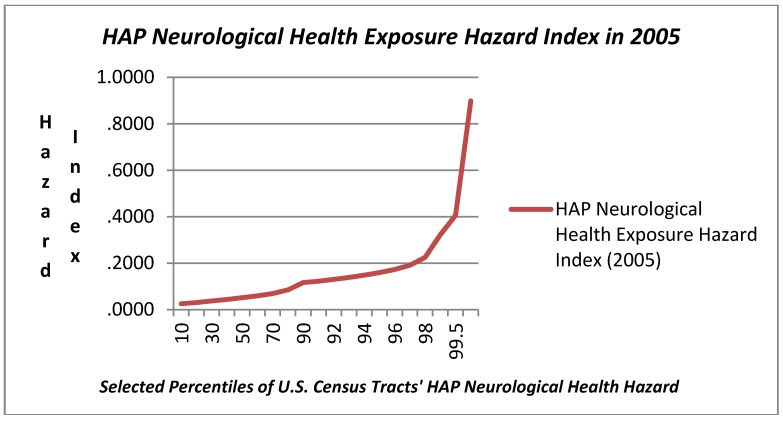
Hazardous Air Pollution (HAP) Neurological Health Exposure Hazard Index (2005) Values at Selected Percentiles for U.S. Census Tracts (U.S. E.P.A. National Air Toxics Assessment, 2005) [44].

**Figure 5:**
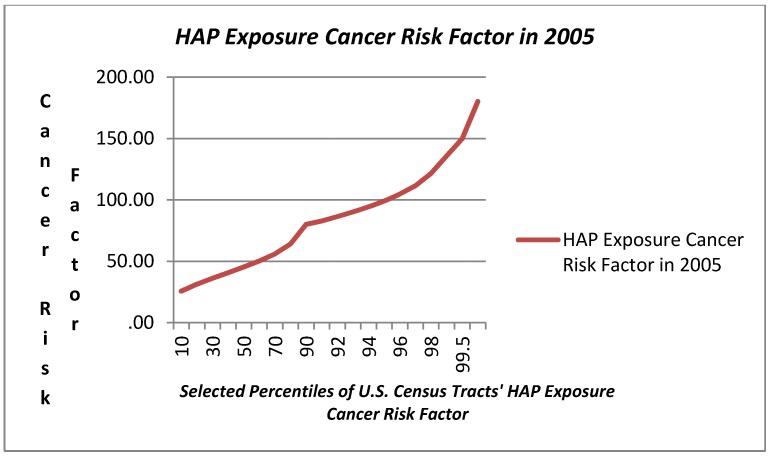
Hazardous Air Pollution (HAP) Cancer Risk Factor Exposure Hazard Index (2005) Values at Selected Percentiles for U.S. Census Tracts (U.S. E.P.A. National Air Toxics Assessment, 2005) [44].

The range in modeled HAP exposure hazard ascertained in the NATA for 2005 related to respiratory, neurological, and cancer exposure hazard metrics is also reported in Table 1 and presented graphically in Figure 3, Figure 4, Figure 5. Respiratory exposure hazard, as modeled by the U.S. EPA in 2005, becomes a magnitude of genuine health risk at around the 75th percentile, and climbs three fold by the 99th percentile. HAP-related cancer risk increases progressively, tripling from the 5th to the 75th, and then increases by 2.3 magnitudes by the 99th percentile. 

**Table 1:** Descriptive Statistics for Study Variables: Tracts & Counties.

Tract-Level Descriptive Statistics (N = 64,524)	Mean	Std. Dev.	Percentiles				
			5th	25th	50th	75th	99th
Population Size of Tracts’ Host County [000s]	1,049.71	1,906.97	16.87	95.79	401.61	1,003.16	9,937.74
Density per Sq Mile Census Tract	5,215.51	11,997.51	17.04	234.18	1,981.92	5,204.29	61,564.29
Townsend Index-Tract	(0.00)	3.08	(3.06)	(2.11)	(0.99)	1.14	10.90
Number Employed in County, 2005 [000s]	436.19	763.05	3.78	32.03	163.77	473.59	3,805.77
Aggregate Wage Payroll (2005) [$000]	19,016	34,796	97	955	5,861	18,893	162,202
Avg. Wages per Employee in County 2005 [$000]	36.05	9.42	23.51	29.26	34.97	40.81	66.37
**County-Level Descriptive Statistics (N = 3,138)**	**Mean**	**Std. Dev.**	**5th**	**25th**	**50th**	**75th**	**99th**
Population Size of Tracts’ Host County [000s]	934.99	3,047.51	29.95	112.28	251.11	636.22	11,745.13
Number Employed in County, 2005 [000s]	364.03	1,330.98	4.77	21.69	65.36	195.73	5,507.85
Aggregate Wage Payroll (2005) [$000]	14,063	64,340	104	524	1,728	5,784	233,483
Avg. Wages per Employee in County, 2005 [$000]	27.71	6.90	19.26	23.44	26.64	30.63	50.26
**Tract-Level Outcomes:**	**Mean**	**Std. Dev.**	**5th**	**25th**	**50th**	**75th**	**99th**
Respiratory Health Hazard (2005)	2.30	1.96	0.44	0.96	1.80	2.99	9.87
Neurological Health Hazard (2005)	0.07	0.10	0.02	0.03	0.05	0.08	0.32
Cancer Risks per MM Population (2005)	50.11	24.38	21.32	33.89	45.39	59.56	135.79

In light of the prominence of exposure in the upper tail of the distribution of exposure among census tracts, the plots in Figure 3, Figure 4, Figure 5 include a range of data points in the highest decile. The three graphs illustrate the very modest gradual increase in exposure hazard values up to the around the 70th percentile, where there is a slight inflexion in the data, and then a steeper rise in exposure until the 90th percentile where there is an exponential rise in the HAP estimates. The exposure curve for neurological health risks of HAPs is clearly different from that for respiratory and cancer HAP exposure hazard in that it is a flatter arc with less variation for census tracts below the 90th percentile. The HAP exposure curve for cancer, as compared with respiratory or neurological health exposure hazards shows a steeper arc in progressive risk from the 10th through the 80th percentile.

### 3.2. HAP Chemical Exposure Hazard by Urbanization and Economic Activity

Table 2 summarizes the data regarding the three forms of HAP health exposure hazard under study and its unadjusted relationship to population characteristics of size, density, and economic activity. Based on a view of unadjusted HAP exposure hazard, there was strong evidence of differential exposure according to county population size and economic activity, and tract characteristics population density and SED. Table 2 illustrates the ratio of HAP exposure at selected percentiles using HAP exposure for the bottom quintile of census tracts nationally as the standard of comparison. We see that the top quintile related to *tracts’ host county* in terms of population size, employment, and payroll per employee, as well as *census tract* population density, has nearly three times the risk reported for the lowest quintile for respiratory HAP exposure. The risk progressively increases from the 80th to the 95th and 97th percentiles as compared to the lowest quintile, indicating a HAP exposure “dose-response” relationship. A similar dose-response pattern is found for the data on HAP cancer exposure hazards, though at lower magnitudes. The data for HAP neurological health exposure hazard do not manifest this dose-response increase in exposure hazard except for census tract population density and SED.

**Figure 6:**
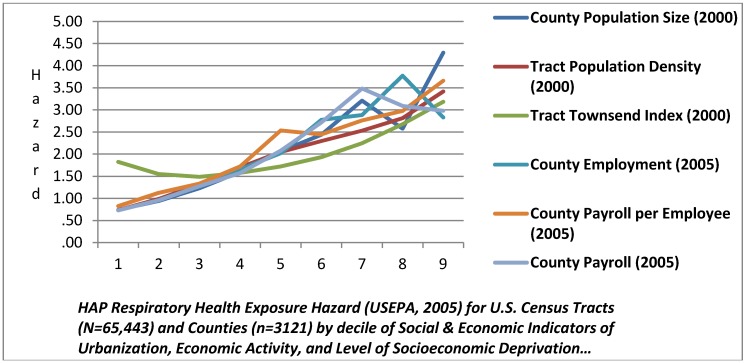
Respiratory Exposure Hazard at Progressive Deciles of Social & Economic Characteristics of Tracts and Counties.

Figure 6 provides an additional view of these relationships by showing curve of the HAP respiratory health exposure hazard index at progressive deciles. We see a positive monotonic relationship between HAP respiratory exposure and county population size up to the 70th percentile, where there is an inflection and decline in exposure between the 70th and 80th percentile, and then a sharp increase in the exposure slope in the top quintile. Tract population density and county economic activity (private business payroll per employee) have positive monotonic relationships with no inflections in the exposure curve. Tract SED is generally flat in respiratory HAP exposure hazard through the 60th percentile, and then climbs exponentially. Similar Figures for Neurological and Cancer HAP data are provided in the Appendix.

**Table 2:** The Social Gradient in HAP Exposure Hazard in U.S. (2005): Risk Ratios of 97th, 95th, and 80th Percentile to the Lowest Decile of Tract Population Size, Density, Economic Activity and SED.

HAP Health Hazard Metrics	Ratio of HAP Exposure Risk at Selected Percentiles to Risk at 20th Percentile	County Population Size (2000)	Tract Population Density (2000)	Tract Townsend Index	County Employment (2005)	County Payroll per Employee (2005)
Respiratory Health Exposure Hazard	Ratio 80/20	2.73	2.85	1.73	3.93	2.65
	Ratio 95/20	2.81	4.51	2.94	4.39	3.23
	Ratio 97/20	5.63	6.3	3.56	5.52	3.64
Neurological Health Exposure Hazard	Ratio 80/20	1.45	2.11	1.57	2.13	1.78
	Ratio 95/20	2.18	3.29	2.59	3.25	1.84
	Ratio 97/20	1.91	4.21	2.82	2.26	1.76
Cancer Risk Factor	Ratio 80/20	1.82	1.85	1.38	2.17	1.83
	Ratio 95/20	1.99	2.63	2.01	2.48	2.11
	Ratio 97/20	3.48	3.08	2.32	3.49	2.11

### 3.3. Differential HAP Exposure Related to Neighborhood SED: Multilevel Model Estimates

Table 3 presents the results from the three multilevel models for predictors of HAP respiratory, neurological, and cancer exposure hazards. SED as measured by the Townsend Index was highly significant and positively associated with all three categories of HAP exposure hazard, after adjustment of county population size and economic activity, and tract-level population density. The cross-level interaction of population size and census tract SED was also found to be highly significant and positively related to respiratory and cancer HAP exposure hazard. In addition, census tract population density was also highly significant and had a strong positive association in all six tests of HAP exposure hazard. County population size and level of economic activity were also highly significant with strong positive associations for respiratory and cancer HAP exposure hazard, however these factors were not found to be statistically significant in the case of neurological HAP exposure hazard. We observed earlier the relatively flat distribution of neurological HAP exposure hazard nationally, and multivariate analysis clarifies that this risk was not significantly related to regional levels of population size and economic activity. The general association of HAP exposure with the East and West coast in the U.S. provides a new perspective in light of the analysis of the regional effects shown in Table 4. The coefficients for the marginal effects of geographic region, after adjustment for county population size and economic activity, and neighborhood population density and SED, reveal a statistically significant and positive regional effect associated with the Pacific coast for respiratory and cancer HAP exposure hazard. In comparison, the coefficients for mid-Atlantic region were not statistically different from zero for respiratory and neurological HAP exposure hazards.

**Table 3:** Multilevel Models of SED & HAP Exposure Hazard After Adjustment for County Population Size and Economic Activity and Tract Population Density: Respiratory, Neurological, and Cancer Exposure Hazards, 2005.

HAP Exposure Hazards	Respiratory	Neurological	Cancer
Tract Population Density (Log 10 Density/Sq M)	0.385 ***	0.0121 ***	5.797 ***
	−0.00538	−0.00057	−0.0687
Townsend Index-Tract	0.0905 ***	0.00312 ***	1.185 ***
	−0.00138	−0.000146	−0.0176
County Population (2000) Log 10	0.247 ***	0.00353	5.947 ***
	−0.031	−0.00334	−0.297
Annual Wage per Employee in Tract’s County ’05	0.0170 ***	0.000331	0.308 ***
	−0.0026	−0.00028	−0.0247
Cross-level Tract SED & County Population Size	0.0138 ***	−2.94 × 10^−6^	0.510 ***
	−0.00079	−8.41 × 10^−5^	−0.0102
New England Region	0.357 ***	−0.00992	1.792 *
	−0.11	−0.0118	−0.983
Mid-Atlantic Region	−0.0684	0.00163	3.617 ***
	−0.0823	−0.00887	−0.746
East North Central	−0.207 ***	0.0132 *	3.057 ***
	−0.0633	−0.00682	−0.59
West North Central	−0.0189	0.000438	4.110 ***
	−0.0609	−0.00655	−0.58
South Atlantic	0.567 ***	−0.00275	10.68 ***
	−0.0607	−0.00653	−0.57
East South Central	0.166 **	0.0217 ***	12.37 ***
	−0.0657	−0.00707	−0.618
West South Central	−0.0179	−0.00828	8.470 ***
	−0.063	−0.00678	−0.595
Pacific Region	0.854 ***	−0.00184	4.812 ***
	−0.0812	−0.00874	−0.749
Constant	−1.372 ***	−0.00616	−21.31 ***
West Region-Intercept	−0.127	−0.0136	−1.21
*Model Fit Statistics:*			
ll_0	−85,224	67,237	−252,964
ll	−76,808	68,045	−240,725
df_m	13	13	13
Number Counties	3,119	3,119	3,119
rho	0.485	0.494	0.294
sigma_e	0.755	0.08	9.706
sigma_u	0.733	0.0791	6.27
chi2_c	67,085	21,898	42,053
ll_c	−110,351	57,096	−261,752
chi2	16,831	1,616	24,477

Standard errors below coefficients; coefficients *** *p* < 0.01, ** *p* < 0.05, * *p* < 0.1.

Although that region was estimated to account for an additional 3 to 4 cancer cases per million related to HAP exposure before adjustment. Statistically significant and high levels of cancer risk were found in the South Atlantic and both East and West South Central regions, after adjustment for population size, density, economic activity and SED.

Evaluation of the goodness of fit statistics for the six models indicate that all share a relatively good fit according to the chi square tests of −2 Log Likelihood, though the weakest model is HAP neurological exposure hazard and the strongest fit is for the HAP cancer exposure hazard. We found relatively strong intraclass correlation coefficients (*rho*) in each model confirming that exposure hazard for tracts within counties are closely related, as was expected, although this was less true for cancer exposure hazard than for respiratory and neurological exposure hazard.

Table 4 profiles the interrelationship of county population size and census tract SED in compounding HAP exposure hazard. These data demonstrate that HAP exposure hazard is not only related to neighborhood SED, but that ***county population size modifies the relationship of SED and HAP exposure.***Effect modification associated with neighborhood SED is illustrated by comparing the three categories of HAP exposure hazard at different levels of SED within varying levels of county population size, holding constant the effect of county economic activity and census tract population density. 

We find dose-response patterns of increased exposure hazard associated with increased levels of SED (20th, 80th, 95th, and 99th percentile) at the each level of county population size—the 20th, 80th, and 95th percentiles. For example, the increase in relative risk of HAP respiratory exposure for a census tract at the 80th percentile of SED compared with the 20th percentile of SED is 26% higher when that tract is in the 95th percentile of county population size as compared with the 80th percentile of county population size. The 99th percentile of SED is associated with 65% higher respiratory HAP exposure hazard when that tract is in the 95th percentile of county population size as compared with the 80th percentile of county population size. This pattern of compounded risk associated with the interaction of SED and county population size is evident for both respiratory and cancer HAP exposure hazard for both years. Thus, there is strong evidence of differential exposure to respiratory, neurological, and cancer-related HAPs related to socioeconomic deprivation, and this differential exposure associated with SED becomes even greater for tracts in the most populous counties in the United States concerning respiratory and cancer HAP exposure hazard.

**Table 4:** Profile of SED Effect Modification of HAP Exposure Risk after Adjustment for County Population Size and Economic Activity and Tract Population Density: Respiratory, Neurological, and Cancer Exposure Hazards, 2005.

Tract SED	Predicted HAP Exposure at 20th Percentile County Population Size			Predicted HAP Exposure at 80th Percentile County Population Size			Predicted HAP Exposure at 95th Percentile County Population Size		
Percentiles:	Resp	Neuro	Cancer	Resp	Neuro	Cancer	Resp	Neuro	Cancer
20th	1.43	0.06	38.80	1.73	0.06	45.75	1.73	0.06	44.01
80th	1.77	0.07	42.65	2.11	0.07	51.04	2.38	0.08	58.91
95th	2.16	0.08	46.91	2.54	0.09	56.89	3.09	0.09	75.36
99th	2.53	0.10	50.97	2.95	0.10	62.46	3.77	0.11	91.04
Ratio 80/20	1.25	1.23	1.10	1.23	1.21	1.12	1.37	1.20	1.34
Ratio 95/20	1.52	1.48	1.21	1.47	1.45	1.24	1.79	1.43	1.71
Ratio 99/20	1.77	1.73	1.31	1.709	1.671	1.365	2.18	1.65	2.07
*Progressive excess risk associated with increased SED, for census tracts at the 95th and 80th percentile of County population size:*							*Case Contrast Assumptions: Mid Atlantic Region; Median Tract Population Density & County Economic Activity; and varying levels of County Population Size and Tract SED*		
				Resp	Neuro	Cancer			
Ratio 80/20				1.122	0.993	1.200			
Ratio 95/20				1.214	0.988	1.377			
Ratio 99/20				1.276	0.985	1.515			

## 4. Discussion

There is extensive public health literature focusing on environmental inequality and environmental justice issues associated with the disproportionate impact of environmental contaminants and air pollutants [5,8,9,37,41,43,46,47,48,49,50,51,52,53,54,55,56]. Neighborhood characteristics relating to SED have been associated with differential exposure to air pollution, as well as related to neighborhood disparities in a range of health outcomes [2,5,14,42,47,49,57,58,59,60,61,62,63,64,65,66,67,68,69]. Neighborhood disparities in SED have been linked as both confounders and effect modifiers with health outcomes related to exposure to PM2.5 and ozone, though little research has been conducted relating to exposure to HAPs [37,60,70,71]. Prior epidemiologic research on SED and air pollution has tended to focus on urban places, with little attention to framing SED and chemical exposure in relation to both rural and urban America, perhaps due to greater availability of data for major cities [62]. Less clear is whether people of lower socioeconomic position were differentially exposed to HAPs across the spectrum of urban and rural places. Prior research has shown SED to be key social epidemiology variables and where there are significant gradients that explain variation in chemical exposure and health outcomes.

The substantive findings in this research are significant in that the SED differential exposure model directly relates increased HAP exposure to a social gradient of neighborhood SED, independent of the regional context of urbanization. The models suggests that there are serious incremental exposure hazards that accrue to progressive levels of socioeconomic deprivation in the United States, independent of urbanization, when we conceptualize urbanization in terms of population size and economic activity. Generalizing from the findings, the risk doubles in magnitude between the 80th percentile and 95th percentile, and is 2.6 greater at the 99th percentile of SED. 

The other contribution of this research relates to cumulative risk assessment methods. It is generally recognized that the inability to quantify risks using comparative metrics accounting for nonchemical stressors—such as socioeconomic deprivation—has limited risk assessors’ abilities to assess cumulative impacts on a consistent, reliable basis, across different locations and time periods and populations. This research illustrates a population health model that enables quantification of the interaction between non-chemical stressors and chemical exposure, including measurement of exposure is modified in a dose-response curve related to SED.

There are two major threats to the validity of these findings. First, the underlying exposure models in the NATA assessments may have misclassified HAPs exposure. Certain regions of the country may be “undercounted” in terms of point source surveillance of facilities. Additionally, the population exposure model may have flaws. Among the most important uncertainties underlying these modeling results are the use of emission estimates from multiple sources (e.g., state- or industry-submitted, computed by EPA from activity data and emission factors, or developed by EPA test programs). According to the U.S. EPA, these estimates differ across geographic regions and source categories, and may vary substantially in quality. Additionally, the exposure model which is applied by the U.S. EPA nationally employs inputs and assumptions that may not be fully representative of particular local areas and the nature of their emissions and concentrations. Among additional caveats for the use of this data is that it projected exposure estimates for the ***median individual within each census tract;***some individuals may have substantially higher or lower exposures than the median individual. The risk estimates are also limited to consideration of inhalation exposure but do not include emissions from indoor sources of air toxics. In addition, people receive substantial additional exposures to pollutants such as mercury, and dioxins that have bioaccumulated in food. The second source of bias is from “built-in” design effects that stem from the administrative organization and long-form sampling of census among tracts which would influence the underlying standard errors of the variables used in the SED measures.

On the other hand, these types of non-random measurement error do not appear to be of the type that would result in a design effect that would confound NATA’s exposure estimates with composite measures of population characteristics of census tracts. On the other side of the argument, there are good reasons for expecting that the magnitude of exposure in inner cities is undercounted given the NATA methodology and what is acknowledged to be excluded.

Given the above limitations, the results of this study demonstrate that HAP exposure was found to be related to the urban concentration of population and economic activity as well as the level of neighborhood socioeconomic deprivation. The size of a city matters as it pertains to both HAP exposure and prevalence of SED. We found relatively strong unadjusted bivariate relationships between HAPs and measures of population density, economic activity, and SED for all three measures of HAP risks in both years examined. SED clearly has two faces in the United States—urban and rural—and there is support for the notion that there are large rural areas with low levels of economic activity, high SED and low HAPs. Multivariate statistical analysis of the geographical distribution of HAPs presents a pattern of exposure hazard that is clearly associated with county population size, the county economy, and localized population density and SED within respective counties. 

Differential chemical exposure among vulnerable populations raises questions about whether we would also find differential response to this exposure. The reference concentration value approach to risk assessment of chemical exposure assumes an underlying uniformity in human physiological response to toxic exposure [35,72,73,74,75]. Two aspects of this situation are worth emphasizing: first, there is clear evidence of differential chemical exposure among higher SED populations; second, the risk metrics that provide the basis for current standards as to what is safe may understate the health risks of HAPs for vulnerable populations. The role and relationship of *nonchemical stressors*, such as extreme poverty or socioeconomic deprivation*,* to multiple chemical exposures therefore represents a key issue in advancing cumulative risk assessment (CRA) and addressing environmental justice issues [35,39]. Social epidemiology is particularly suited to study differential exposure and differential susceptibility related to the interaction of chemical and nonchemical stressors in cumulative exposure and risk assessment.

## Figures and Tables

**Figure A1:**
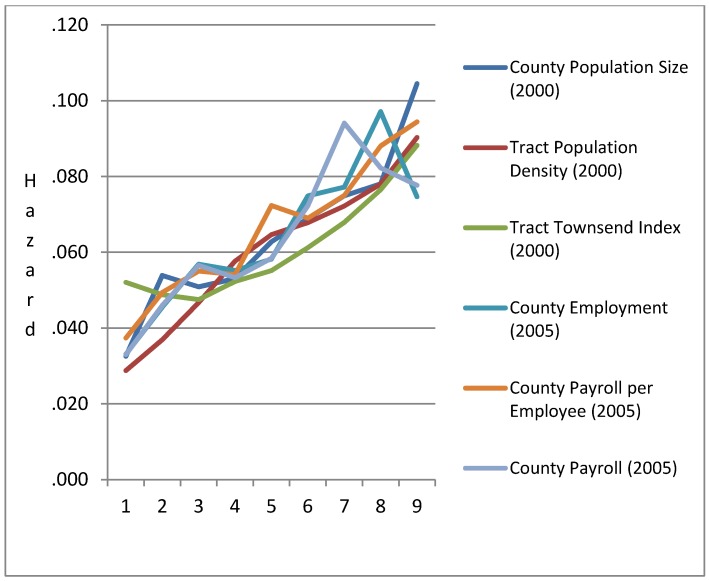
Gradients in HAP Neurological Health Exposure Hazard (2005) at Progressive Deciles of Population Size, Density, Economic Activity, and Socioeconomic Deprivation.

**Figure A2:**
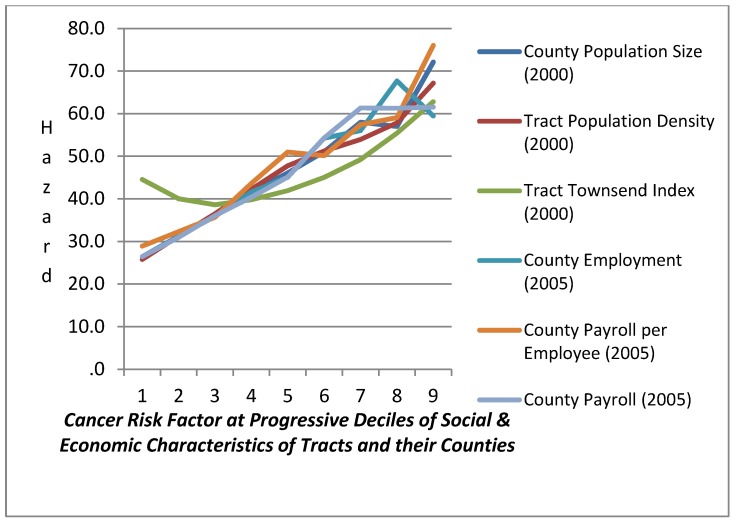
Gradients in HAP Cancer Exposure Risk per MM (2005) at Progressive Deciles of Population Size, Density, Economic Activity, & Socioeconomic Deprivation.

## Appendix

**Table A1:** Study Population: U.S. Census Tracts and Counties (2000).

Census Division:	Counties (Pct)		Population (Pct)		Tracts (Pct)	
New England	67	2.1	14,238,888	4.8	3,203	4.9
Middle Atlantic	150	4.8	40,332,259	13.7	9,918	15.2
East North Central	437	13.9	46,031,860	15.7	11,328	17.4
West North Central	618	19.7	19,697,992	6.7	5,090	7.8
South Atlantic	589	18.8	55,182,959	18.8	10,781	16.5
East South Central	364	11.6	17,480,032	6.0	3,938	6.0
West South Central	470	15.0	33,281,974	11.3	7,104	10.9
Mountain	281	8.9	19,823,587	6.8	4,255	6.5
Pacific	165	5.3	47,610,448	16.2	9,549	14.7
Total	3,141	100.0	293,679,999	100.0	65,166	100.0
